# From good to great: The journey of sustained academic improvement of a university department of pathology and laboratory medicine

**DOI:** 10.1016/j.acpath.2025.100159

**Published:** 2025-02-03

**Authors:** Zu-hua Gao, Genevieve McMillan, Cheryl Wellington, Helene Cote, Michael Nimmo, David Huntsman, Suzanne Vercauteren, Lucy Perrone, David Granville, Cornelia Laule

**Affiliations:** Department of Pathology and Laboratory Medicine, University of British Columbia, Vancouver, BC, Canada

**Keywords:** Budget, Engagement, Education, Governance, Research, Strategic plan

## Abstract

Pathology and Laboratory Medicine discipline occupies a special place in the medical school and healthcare ecosystem as it bridges basic medical science and clinical practice. In the era of rapid knowledge and technology evolution, the new ways of communication, new training requirements, and the demand of personalized precision diagnostics, this specialty is facing unprecedented challenges and opportunities. Some of these challenges are institution-specific, while many are shared worldwide at different magnitudes. This review shares our team efforts in effectively dealing with challenges of budget constraints, engaging diverse and distributed faculties to establish and implement a shared vision and strategy to achieve sustained improvement in education programs and research enterprise. We hope that our experiences and insights can inspire other university departments in finding innovative approaches for their unique challenges and opportunities.

“*Greatness is not a function of circumstance. It is largely a matter of conscious choice.* ”--Jim Collins^1^

On the international level, there is substantial variation in how university departments of pathology and laboratory medicine are structured and positioned within the university and the healthcare ecosystems. Some departments consist of only anatomical pathology, others are composed of both anatomical and clinical pathology; the latter include microbiology, biochemistry, cancer cytogenetics, hematopathology and transfusion medicine, etc. All university departments have the mandate of research and education that includes classroom teaching, research laboratory training, and clinical on-site teaching of undergraduate, graduate, and postgraduate trainees. However, the involvement in clinical operations and the engagement of clinical faculty varies significantly. Some challenges such as limitation of budget, space, and resources are universal. Other challenges are unique to each institution. This article intends to share our experience with effectively managing our unique challenges, and achieving sustained improvement of a large and complex academic department of pathology and laboratory medicine at the University of British Columbia (DPLM-UBC).

## Background

DPLM-UBC is the only academic pathology and laboratory medicine department in British Columbia, a province of 5.5 million people. The department consists of 48 tenure-track research faculty members, 313 clinical faculty members, and 178 research staff. In addition to contributing medical school undergraduate teaching (151 students), the department has its own undergraduate bachelor of medical laboratory science program (BMLSc, 40 students), graduate program (69 students), 5 resident training programs (anatomical pathology, hematopathology, neuropathology, medical microbiology, and medical biochemistry, 39 residents), clinical fellowship programs (14 fellows), infection prevention and control certificate program (21 students), laboratory quality and proficiency testing program (113 students), and multiple continued medical education programs (e.g., Canadian Anatomical and Molecular Pathology courses, Pediatric & Perinatal Pathology Slide Club, etc.). Clinical faculty members in the department are located at 6 affiliated hospitals (Vancouver General Hospital, St Paul’s Hospital, BC Cancer, BC Children’s and Women’s Hospital, BC Center for Disease Control, and Royal Columbian Hospital) and teaching hospitals in seven health authorities (Northern Health, Interior Health, Island Health, Vancouver Coastal Health, Provincial Health Services Authority, Fraser Health, and First Nations Health Authority). The department is the home of 5 Fellows of the Royal Society of Canada, 7 Fellows of the Canadian Academy of Health Sciences, and 6 active Canadian Research Chairs (CRCs). In addition, there are also 26 affiliate, honorary, adjunct, and visiting professors from other departments or other institutions. Faculty members participate across a spectrum of research from basic investigative to translational to clinical applied research and education scholarship; they are recognized locally, nationally, and internationally for their excellence. Collectively, faculty members in the department generate greater than 9% of all research funding in the Faculty of Medicine (FoM). The areas of recognized strength include but are not limited to cancer, molecular pathology, genomic science and bioinformatics, cardiovascular and pulmonary diseases, neuroscience and related diseases, blood-related disorders, diabetes mellitus, and microbiology/virology. Faculty members and administrative staff of the department are very active in academic services for the department, the FoM, UBC, and external clinical or academic organizations, and often hold key leadership positions. Based on the research performance among 5779 university pathology departments, DPLM-UBC is ranked #28 in the world, #24 in North America, and #2 in Canada.^2^

In his fact-finding journey immediately after coming on board in November 2021, the new head of DPLM-UBC started meeting with more than 100 key stakeholders within and outside the department, toured different hospital sites, and met with clinical faculty members at hospital sites as a group. On the departmental level, the questions asked were: What is working? What is not working? What are your suggestions? On the personal level, the conversation was focused on the understanding of each individual’s accomplishments (strength), priorities, plans, challenges, concerns, and needs, and how can the department help them to accomplish their career goal. This on board consultation exercise has helped to gain a more complete understanding of the department’s unique strengths, weaknesses, challenges, and opportunities, in addition to universal challenges and opportunities of all academic departments.^3^ Specifically, three major interconnected unique challenges were identified: (1) due to the fact that the university does not fund merit increases, performance salary adjustments and career progress increments and that most if not all overheads on grant funding go to the research institutes, in addition to other factors, the department has a large structural deficit that is expected to increase every year. As a result, we are unable to develop a succession plan for several internationally renowned research scientists and academic leaders who are expected to retire within a decade or so. Without effective succession planning, the department has a genuine risk of substantial reduction of its academic capacity and impact in the foreseeable future; (2) significant effort and investment are required to ensure our education programs are competitive and keeping up with the rapid knowledge evolution, the new ways of communication, new training requirements, and the changing needs of the job market; (3) clinical laboratory physicians at distributed hospital sites across the province lack a sense of unity and belonging to the UBC department. DPLM-UBC has been considered by many people to be a university *“PhD-only*” department.

## Model governance

**Fig. 1:**
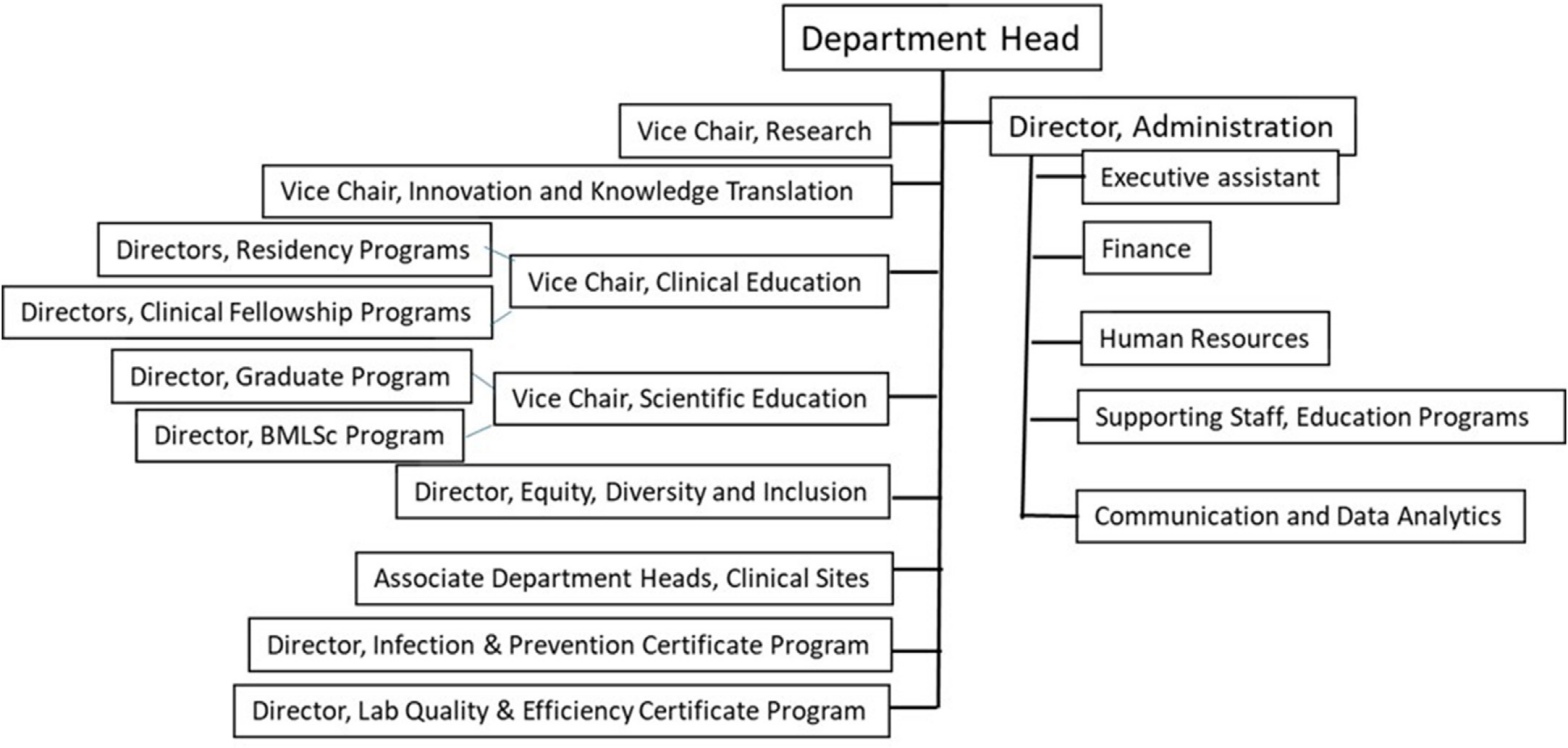
Departmental organization structure.

The department is privileged to have some extremely talented visionary academic leaders who are passionate about the well-being of the department and its academic programs. Taking advantage of this talent pool, and through an open competitive process, we appointed a vice chair of research, a vice chair of innovation and knowledge translation, a vice chair of clinical education, a vice chair of scientific education, a director of equity, diversity, and inclusiveness (EDI), and a director of external relations. We also appointed an associate academic department head in three health authorities. Each vice chair meets with the department head every other week, and all serve on the department’s executive committee which meets bimonthly. This allows the department head to have time to focus on strategic issues, while at the same time being on top of major activities of the department. Critical issues are discussed, and strategic decisions are made collectively by all senior leaders of the department during executive committee meetings. This team leadership model has proven to be effective in defining and implementing the strategic priorities and avoiding the risk of making major mistakes (Fig. 1).

At the department level, we have constructed the following standing committees with regular meetings for managing the ongoing departmental business: departmental executive committee; clinical appointment, reappointment and promotion committee; academic appointment, reappointment, promotion and tenure committee; finance committee; human resources committee; awards committee; summative peer review of teaching committee; merit committee; pathology day planning committee; program directors executive committee; communications committee; province-wide rounds committee; etc. Each education program has its own committees with regular meetings such as the resident training committee for each resident training program, the BMLSc management committee, the graduate and post-doctoral studies committee, the graduate program curriculum committee, etc. In addition, we will form temporary cross-cutting committees for managing the timely sensitive new business such as the faculty renewal selection committee, etc. Committee memberships were structured following EDI principles. All committee meetings are run following Robert’s rules of orders and decisions are made through consensus.^4^

## Strategic planning

The new department head’s extensive engagement with faculty in his first 100 days in office set the stage for an inclusive strategic planning process, launched officially with the recruitment of a 17-person steering committee, broadly reflective of the many different groups of the department, including learners and staff. The kick-off meeting framed and anchored the process in the requirement for alignment with the strategic plan of the FoM.

Based on the initial data gathered by the department head and presentations by the vice chairs of research and education, opportunities in front of the department were identified, together with the issues to be resolved, and the engagement approach to be followed. Interviews were then conducted with key external and internal stakeholders, the results of which informed a survey designed to obtain further input from external stakeholders, faculties, learners, laboratory and administrative staff. Results were shared and discussed during the DPLM-UBC weekly province-wide rounds that led to the identification of four strategic directions together with proposed strategic priorities.

A deeper, more focused phase was launched three months later, with the chartering of five lead groups, two for education (scientific and clinical), one for collaboration, one for partnerships, and one for organization/people. These groups involved some 80 faculty members, learners, and staff, and met on more than thirty occasions. The results of these discussions served as vital inputs to an all-day in-person steering committee meeting where the strategic direction was finalized, and strategic priorities articulated.

We first clarified our collective values as “global relevance, societal commitment, integrity, collegiality, curiosity, innovation, equity, and inclusiveness”. Based on these set of values, we defined our mission as “We are an inclusive department of pathology and laboratory medicine where cutting-edge discoveries are made, future leaders in medicine are trained, and patients receive the highest quality of care.” We jointly crafted our vision as “Transform laboratory medicine and our understanding of disease for better health”. Our vision is in complete alignment with that of the FoM “Transform health for everyone.” We developed our strategy and action plan around four set of goals: research, education, collaboration, and organization.^5^ A detailed implementation plan outlining the department’s priorities and individual pillar priorities has been developed, identifying individual accountabilities where appropriate. Progress against these plans is discussed as part of the regular monthly meetings between the department head and each vice chair and has become a standing item on the executive committee meeting agenda.

On an annual basis, the department head and vice chairs will provide a progress report outlining achievements and highlighting the successes of the department. Priorities for the following year, within the context of the 36-month timeframe identified, will be discussed by the executive committee, and put forward for consideration by faculty at the department’s annual planning day. As part of that discussion each year, both external and internal forces and trends will be considered. The external environment is dynamic and volatile which can necessitate making different choices over time.^6^

This strategic planning process has proven to be effective in improving faculty engagement, departmental culture, and staff morale, and enables us to align all our energy and resources for a shared vision and a set of common goals.

## Balancing the budget

Pathology and laboratory medicine are a specialty that does not have direct contact with the patient population we serve, which poses a huge challenge to raise funds through philanthropy. Innovative approaches are needed to improve societal awareness of the important clinical contributions of our clinical faculty members and the importance of scientific discoveries made by our research faculty. Following the FoM’s relationship with industry policy,^7^ we engaged industry partners and obtained sponsorship to cover the cost of external speakers for our annual Pathology Day event and our weekly province-wide rounds. Working with the FoM Development Office, we created donation buttons on our departmental website to facilitate donations and their targeting to specific areas. This initiative has proven to be an effective tool to raise funds to support our academic activity.

**Fig. 2:**
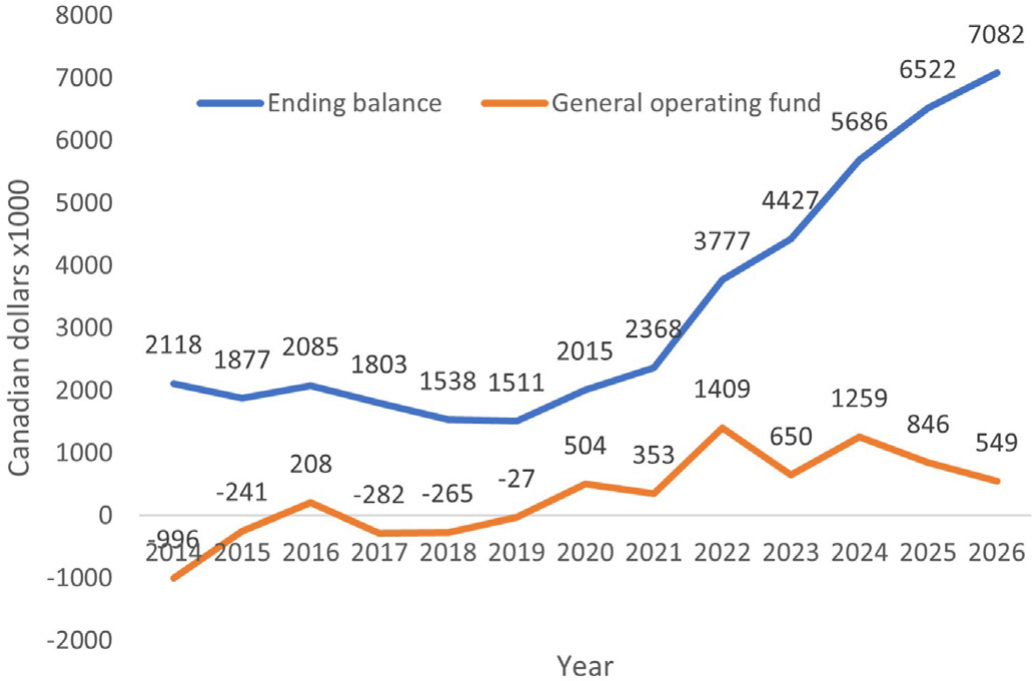
Starting from a structural deficit in the general operating fund in 2014, there is a sustained continuous improvement of the department’s general operating fund and the ending balance. In January 2025, we will start the clinician scientist program.

Salary and annual salary increase of academic faculty members including those related to performance salary adjustments, merits, and career progress increments occupy the biggest portion of the departmental budget. To offset this cost, we increased the number of tier-1 active Canadian Research Chairs from 3 to 6, each with a significant contribution of the salary for up to 14 years. Working with hospital foundations and research institute funding partners, we raised funds to cover a significant portion of salary for some of our junior faculty members. We had the courage to end some of the academically non-active appointments and processed some retirements of our senior faculty members. We streamlined operational processes and cut some unnecessary IT costs. We increased the number of international trainees in our education programs such as the graduate program, laboratory quality and proficiency testing, and clinical fellowship programs. We established a grant-writing practicum course and revamped our infection prevention and control course; both have the potential to generate more funds for the department. Thanks to the collective effort of our administration director, our finance team, and our academic leaders, we were able to significantly reduce our structural deficit, achieved a healthy budget in our 10-year financial forecast, and have sufficient funds to plan for succession of our projected retirements, and establish a flagship clinician scientist program (Fig. 2).

## Engagement and relationship building

### Engaging members within the department

In addition to the university campus, our research faculty are geographically located in multiple research centers/institutes (BC Cancer Research Institute, BC Children’s Hospital Research Institute, Centre for Blood Research, Life Sciences Institute, Djavad Mowafaghian Centre for Brain Health, Centre for Heart Lung Innovation, Providence Research, Vancouver Coastal Health Research Institute, Women’s Health Research Institute, Prostate Center, Ovcare, International Collaboration on Repair Discoveries, etc.), and our clinical faculty members geographically located in hospitals of seven health authorities across the province. To engage all members within the department across the province, we established weekly province-wide rounds with topics alternating among research, clinical diagnostics, leadership, and departmental business. The department produces biweekly news bulletins, semiannual departmental newsletters and an annual report to the community. We organize an annual “Pathology Day” event with both external and internal keynote speakers and showcase the research work by our trainees in a format of oral and poster presentations. Multiple departmental level awards were given at both Pathology Day and a seasonal celebration party to acknowledge the excellent contributions of both academic and clinical faculty members, as well as trainees and staff members. Our resident program and our graduate program both have an annual graduation ceremony. Both the BMLSc and graduate programs have initiated annual alumni events. The department head scheduled annual individual meetings with all academic faculty members and visits clinical sites to meet clinical faculty members regularly.

### Building relationships across a range of stakeholders

**Fig. 3:**
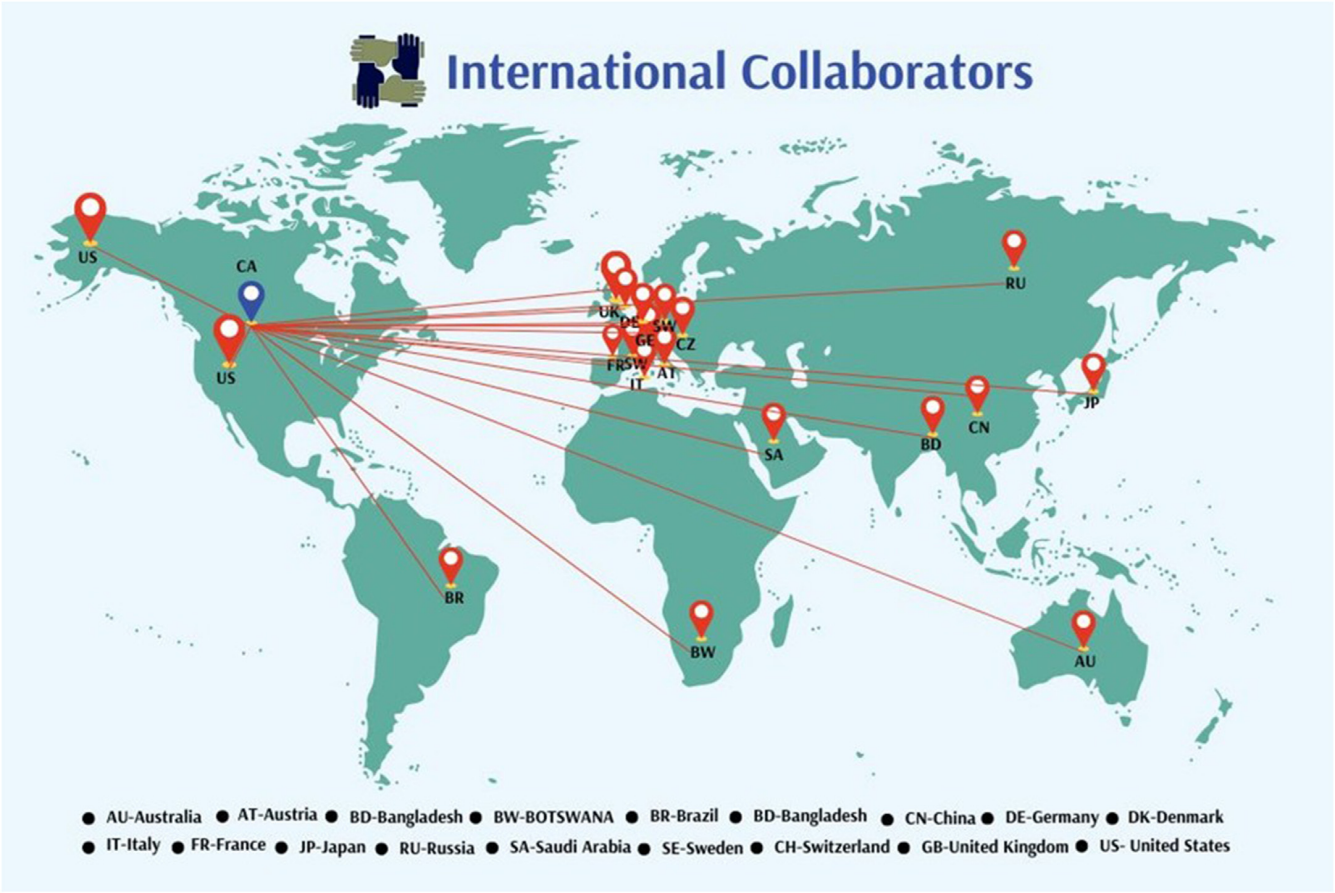
Clinical and academic faculty members have established research collaborations with colleagues in 19 different countries.

Within UBC, we worked with other academic departments and schools (e.g. computer science, biomedical engineering, medicine, cellular and physiological science, etc.) for cross-disciplinary education, research, and joint recruitment. Within the health sector, we worked with the provincial ministry of health, clinical department heads, and provincial medical laboratory services (PLMS) to ensure alignment and integration of research, education programs, and clinical activities. The UBC department head and the vice chair of clinical education attend clinical department meetings of St Paul’s Hospital, BC Centre for Disease Control, BC Children’s and Women’s Hospital and BC Cancer. The vice chair of clinical education is a senior leader of Vancouver General Hospital department of Pathology and Laboratory Medicine. The UBC department head sits on the senior leadership committee of PLMS and the PLMS executive director sits on the UBC department executive committee. PLMS and UBC-DPLM have established regular joint business meetings. In addition to spending 20% of the time doing consultation work on cancer pathology, the UBC department head assumed the clinical leadership role as the provincial program medical director for pathology and laboratory medicine at BC Cancer. The credibility established by doing clinical work and clinical leadership helps the UBC department head to better engage with the clinical community. To deepen relationships with the biomedical community to foster understanding of their skill needs and to speed up the translation of research into practice, we organized monthly “BioConnect BC” events to bring together faculty, clinicians, scientists, residents, trainees, staff, entrepreneurs, bio/pharm staff/execs/veterans, lawyers, venture capitalists, and others with common interests in biomedical/translational research and discovery/commercialization to casually interact. On the international level, our province-wide rounds and pathology day features internationally renowned speakers. We established a “visiting scientist” program to allow regular visits of internationally renowned scientists and educators to our department. Research scientists in our department are involved in over 100 global research initiatives. International collaboration activities help to facilitate the establishment of new research networks and ensure our trainees and faculty members are constantly informed of the new scientific advances, and at the same time improve the global awareness and impact of the work done by our faculty members (Fig. 3).

## Revitalization of education programs

Over the past few years, our departmental signature BMLSc program has experienced important challenges. The number of applicants enrolled to the program decreased to 14/year (capacity of 24/year). Under the leadership of vice chair of scientific education, we first organized a committee and reviewed the program internally. Taking the suggestion of all key stakeholders, we organized a team to upgrade our teaching curriculum to reflect new technological advancements and the needs of the job market. We established a co-op program to facilitate exposure of students to the work and research environments and improve their employment competitiveness. We facilitated in-house events to introduce extra-curricular opportunities by collaborating with other units on campus (e.g. Go Global, Career Services, and Enrolment Services). Thanks to the extraordinary effort made by the team led by the vice chair of scientific education and the new program administrator, for September 2024 the number of applicants enrolled to the program has returned to full capacity with 24 or 25 students, including two indigenous students.

**Fig. 4:**
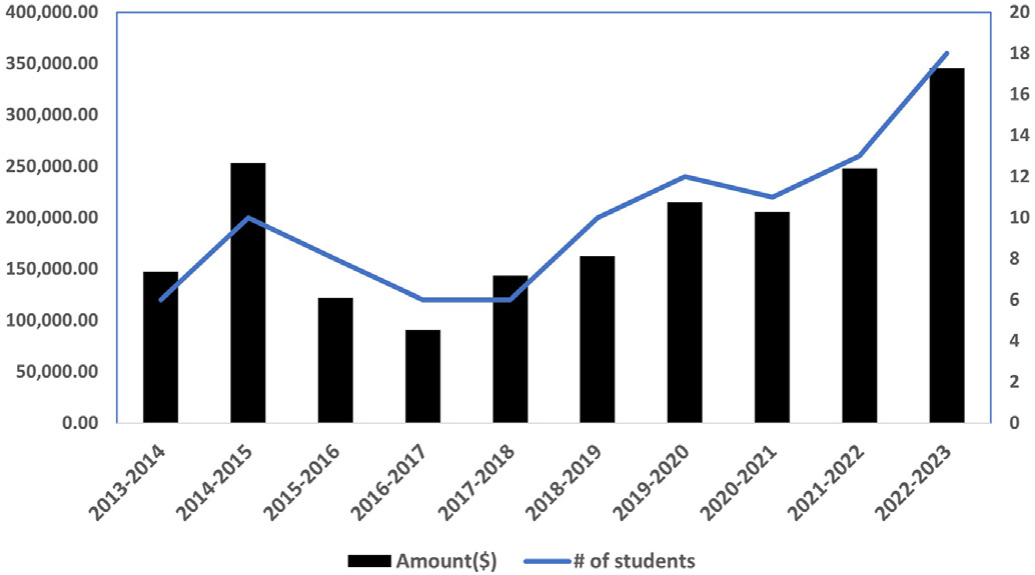
Increased dollar value of awards and the number of graduate students who won the Canadian Institute for Health Research awards each year.

To ensure our graduate curriculum is competitive and kept abreast of the rapid advancement in science and technology, the program director held a round table consultation about curriculum needs. Following the consultation, the program developed several new courses and updated existing curriculum to include teaching around Big Data analysis and visualization, machine learning, genomic and single cell analysis, and the use of R. We also surveyed broadly with student and supervisors in the DPLM-UBC graduate studies program and revised the format of the DPLM-UBC comprehensive exam to allow students to develop grant application ideas more closely related to their thesis project. We established an alumni engagement committee and held the first alumni event. The DPLM-UBC Student Association (PaSA) organized several professional development activities and social events for graduate students and post-doctoral fellows. Trainees in the graduate program received several external awards including a Vanier award, 4 CGS-Doctoral and 4 CGS-Master awards. Together, as of April 2024, the graduate students in the DPLM-UBC graduate studies program have received over $2.75 million in competitive awards/scholarships over the course of their graduate studies (Fig. 4).

**Fig. 5:**
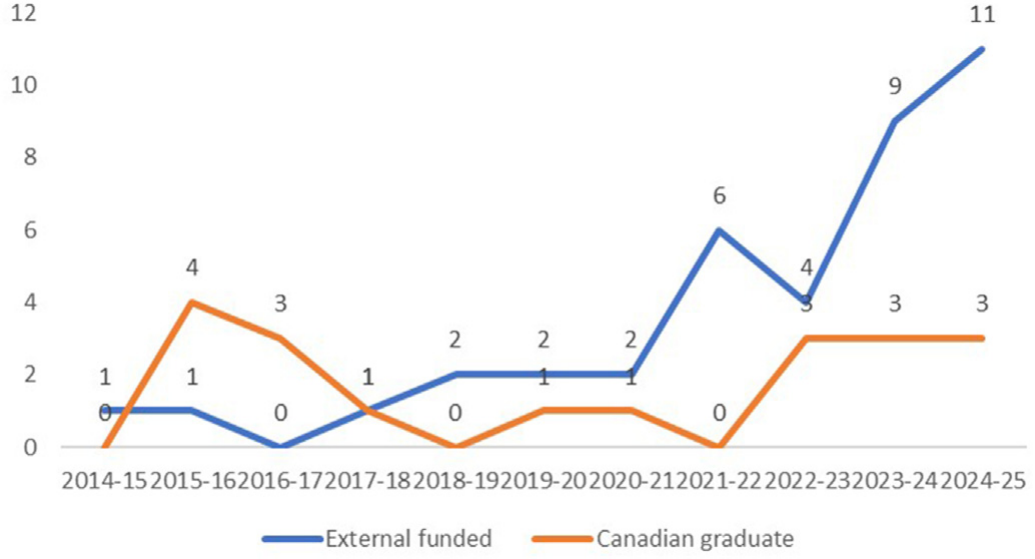
The department had a significant increase in the number of clinical fellows, particularly those externally funded fellows in the past 3 years.

To ensure our postgraduate trainees are not only able to successfully pass the Royal College licensing exam but also ready to practice in the community with updated knowledge and skills, our postgraduate education team led by the vice chair of clinical education and the program directors has implemented several innovative strategies. For junior residents in their first 3 years, we created a series of on-demand digital sessions on practical approaches to common entities in each subspecialty. We established multiple community rotation sites so that our residents can have sufficient exposure to the real-life cases in the community. For senior residents in their 5th year of training, we created an Area of Special Interest (ASI) training program, which is a series of consecutive blocks in one subspecialty equivalent of a mini-fellowship experience. We worked with the university postgraduate office to ensure that a resident who is successful at completing an ASI will have this added designation placed on their university graduate diploma. In the past 5 years, 37 residents graduated from our department with 100% passing rate of the Royal College licensing exam. In addition, there was a significant increase in the numbers of clinical fellows, particularly external funded clinical fellows in the past 3 years, which further testifies the quality and international reputation of our post graduate training programs (Fig. 5).

Our consultations with department members during the strategic planning process identified the need for a Pathology Assistant Master’s Program as well as for a fellowship program for clinician scientist program. PLMS facilitated a province-wide needs assessment for a pathologist’s assistant program. The survey result guided us to focus on establishing the educational programs as part of our educational initiative. We started revamping the certificate course in Infection Prevention and Control into a 25-week online course that will help the health authorities prepare new infection prevention and control professionals. In 2023, our Canadian Microbiology Proficiency Testing program graduated and certified 58 students, supported point of care testing in 151 sites in 12 provinces including 6 international sites. Our laboratory quality program graduated and certified 55 students and hosted an international lab quality conference with 116 attendees from all over the world.

## Strengthen the research profile

**Fig. 6:**
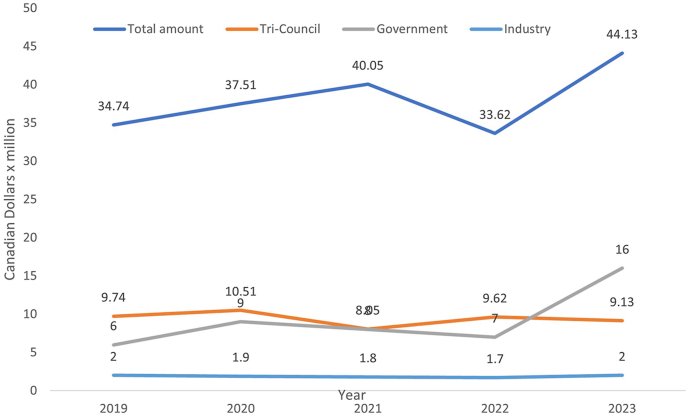
In the last five years, research funding received into the department has increased from $34.7 million to $44.13 million dollars per year. Most of this increase is due to an increase in government funding and other non-profit agencies. The data also show stable tri-council grants funding.

Led by the vice chair of research and her team, we established our strategy around the goal to create, translate, and implement knowledge across the research continuum that promotes improved individual and population health. We launched our inaugural pathology grant application practicum, a 4-month didactic interactive workshop covering a variety of topics in how to prepare, generate and evaluate competitive grant proposals. Sessions were tailored for early career faculty, clinical faculty new to research, and senior trainees in a transitional role. Workshop participants could choose to submit a seed grant application or serve in a grant application reviewer role. We also established an interactive research dashboard to track individual investigators’ research grant successes over a 10-year period. This dashboard helps us to identify strengths and weaknesses in research across our department and allocate resources for targeted support. We have successfully gathered information about biobanks held by our departmental faculty members, enabling us to explore methods to increase access to and use of human tissue and fluid specimens. We collected information on research interests, available equipment, and trainee supervision, enabling better searchability of faculty members through our departmental website. Although there has not been a significant increase in the government Tri-council (Canadian Institutes of Health Research, Natural Sciences and Engineering Research Council, and Social Sciences and Humanities Research Council) funding, in 2023 our overall research funding has reached a record high of 153 competitive research grants with a total value of $44,131,444 dollars in 2023 (Fig. 6). In 2023, our departmental members published over 620 peer-review articles, won 32 awards, and obtained 22 patents.

## Equity, diversity, inclusions, and indigenous reconciliation

The goal of DPLM-UBC is to create an inclusive and safer spaces to engage indigenous, black, racialized, and equity-deserving students, staff, faculty, and members of the clinical community. The department's Inclusion Action Committee conducted an EDI survey and received more than 200 responses, which provided us with a concrete understanding of reality and the gaps that needs to be filled. In response to the survey, we implemented a policy that there will be an open call and selection process for all leadership positions in the department. We repurposed one of our endowments to create a bursary to address economic inequity among our trainees. We embedded anti-racism, anti-oppression, and the recommendations from the Truth and Reconciliation Commission into our curriculum and training of our students. We adopted EDI principles in our recruitment, promotion, and award processes. EDI training has become mandatory for all faculty members who serve on the academic recruitment committee in our department. We require our faculty members to report their EDI-related activities in their annual self-assessment report, and EDI is an important part of their performance evaluation.

A member of our faculty established a summer training program in science, technology, engineering and mathematics (STEM) for Indigenous youth called seed2STEM. The program invites high school students from grades 9 to 12 to participate in paid, six-week summer research internships covering various STEM topics. In addition to earning minimum wage for working 25 h per week, students engage in weekly research-focused learning modules, hear from guest speakers, and visit research labs, local scientific and cultural sites on field trips. With the support from the UBC’s STEM funding, the Faculty of Medicine’s Strategic Initiative Fund and private donations, the seed2STEM started in 2018 with one student and expanded to 17 students in 2023.

We have also focused on the diversity of our academic faculty. With the new recruitments in the past 3 years, we have increased the percentage of female assistant professor from 30% to 43%. In 2023, the department sponsored three candidates from underrepresented ethnic groups in their application to the Canadian Institutes of Health Research Respect, Equity, Diversity and Inclusion Early Career Transition Award. The successful candidate was subsequently offered a faculty position in the department. We submitted a nomination for a Canadian Research Chair Tier II for young academics who have a disability. As mentioned earlier, two indigenous students are admitted into the BMLSc program in 2024. In the past 5 years, there have been 5 academic recruits, 48 clinical recruits, and 3 clinical faculty selected for protected research time with the goal of conversion to an academic appointment. With the joint effort of the department and the FoM, we were able to successfully retain three prominent professors who have received a competitive offer elsewhere. The reports from the 2024 external review indicates that all academic and clinical faculty members in the department are pleased with the progress made in the recent years and the open, transparent, collegial, and inclusive working environment.

The DPLM-UBC award committee added an award for EDI to its awards. It recognizes the efforts of faculty members, learners, staff, post-doctoral scholars, and staff who contribute to an equitable, diverse, inclusive culture in the DPLM-UBC. The first award was presented at the Pathology Day, 2024. These efforts have effectively changed the EDI landscape of the department and set an inclusive departmental culture. In 2023–2024, our dashboard showed that female and male academic faculty members have the same success rate at obtaining grants.

## Built to last^8^

As Phillip C. McGraw once said, “Life is a marathon, not a sprint.” To achieve sustained continuous improvement, we have employed the following three mechanisms:

1 Clinician scientist program. Through a competition process, the department selects 3 to 5 early career clinicians each year and provides $50,000 per year for 3–5 years. With the joint in-kind support of the clinical department head, the intention is for those successful candidates to have at least 20% time protected for research and teaching. Among this cohort of clinician scientists, we will identify stars who show evidence of obtaining external grant funding and establishing a successful academic career. For those star clinician scientists, the department will work with hospital foundations and other sources to provide continued support for their academic activities. This clinician scientist program will help the DPLM more closely engage the clinical community and have a pipeline of academic physicians to sustain and expand the academic capacity of the department.
2 Road map for PhD scientist succession. We established a departmental research succession planning committee. The committee’s task includes scanning the environment to determine what is the future of biomedical science and where we have potential to have strategic partners that can provide financial and infrastructure support. The department head, the administrative director, and the finance director worked together with the succession planning committee to establish a realistic road map for PhD scientist succession. This structured forward-looking approach ensures the sustainability and enhancement of the internationally leading research programs in the department.
3 Annual celebration and re-planning. As discussed in the strategic planning section above, each year in September, academic leaders of the department and key stakeholders will gather in a retreat format. During the retreat, we will first reflect and learn by celebrating our success and analyzing the causes of unsuccessful initiatives. Then we will re-scan the environment, identify new opportunities, and plan new initiatives for the next year. This yearly structured approach will not only sustain our existing strength but achieve continuous improvement in our academic deliverables.

We have shared our experiences in successfully dealing with our unique challenges such as elimination of the structural deficit, engagement of clinical faculty, partnership with the health authorities, the industry and international academic community, improvement of our educational programs, and increasing our research funding and output. Our innovative structured approach has proven effective in achieving sustained improvement of our academic mandates. We hope that our experiences and insights can inspire other university departments in finding innovative approaches for the unique challenges and opportunities they face.

## Funding

This research received no specific grant from any funding agency in the public, commercial, or not-for-profit sectors

## Declaration of competing interest

The authors declare that they have no known competing financial interests or personal relationships that could have appeared to influence the work reported in this paper.
